# Editorial for Special Issue “Mental Disorder: Focus on Pathogenesis to Treatment”

**DOI:** 10.3390/cimb47100862

**Published:** 2025-10-18

**Authors:** Fabrizio Bella, Cecilia Chiarenza, Carmen Concerto

**Affiliations:** Psychiatry Unit, Department of Clinical and Experimental Medicine, University of Catania, 95123 Catania, Italy; faber.bella92@gmail.com (F.B.); cecilia.chiarenza11@gmail.com (C.C.)

In recent years, advances in molecular biology have enabled the investigation of previously inaccessible mechanisms at the cellular and immunological levels that underlie the pathogenesis of numerous conditions affecting the central nervous system. Mental disorders, which remain far from being comprehensively defined in pathophysiological terms, continue to pose a major challenge for both clinicians and researchers. Formerly conceptualized as mere epiphenomena of aberrant neurotransmitter production, they are now recognized as complex and multifactorial disorders of large-scale brain networks. Their pathophysiological characterization increasingly requires integration of both genetic and epigenetic dimensions, a prerequisite for the development of tailored therapeutic strategies and valuable biomarkers. Emerging evidences have more clearly delineated the role of neurotrophins in neuroplasticity, together with their receptors and inducible factors, with important implications for elucidating the pathophysiology of Major Depressive Disorder (MDD) [1]. Likewise, increasing attention has been directed toward inflammatory pathways within the central nervous system, which appear to play a pivotal role in the development of affective disorders and conditions within the Schizophrenia spectrum. These insights have been made possible by the evaluation of gene expression profiles associated with key effectors of the immune response [2].

At a more refined level, recent investigations have sought to unravel the complex regulatory networks governing gene expression. In particular, brain-derived non-coding RNAs—including long non-coding RNAs, microRNAs, and Y RNAs—have emerged as critical modulators. These molecules, which can also be detected in circulating exosomes from peripheral blood, exert several functions ranging from the regulation of their host genes to scaffolding and gene-silencing activities across distant genomic loci [3].

Beyond pathophysiological inquiry, the applications of molecular biology in psychiatry are increasingly shaped by advances in molecular biotechnologies. Of particular relevance is the expanding field of gene therapy, which holds the promise of deleting, silencing, or editing defective genes and delivering genetic material capable of producing therapeutic molecules [4].

The aim of this Special Issue entitled “Mental Disorder: Focus on Pathogenesis to Treatment” is to gather the knowledge about the ongoing advances in the biomolecular dimensions of psychiatric disorders worldwide. It includes five studies, distinguished by their novelty and by the potential directions for future investigation that the authors’ work has helped to delineate within this compelling field of research.

The study “Peripheral Blood Exosomal miR-184-3p in Methamphetamine Use Disorder: Biomarker Potential and CRTC1-Mediated Neuroadaptation” from Zhao et al. [5] investigates the role of circulating exosomal miRNAs in methamphetamine use disorder (MUD). Research on non-coding RNAs represents a hot topic in molecular neuroscience, characterized by significant challenges, continuous innovation, and promising opportunities for discovery. Authors found that miR-184-3p (and to a lesser extent miR-4433a-5p) is significantly reduced in MUD patients compared to controls. ROC analysis shows that miR-184-3p has high diagnostic accuracy (AUC up to 0.902), making it a potential peripheral biomarker for MUD. Moreover, miR-184-3p regulates the CRTC1 gene, which is involved in neuroadaptation through the CRTC1/CREB pathway, suggesting a possible biological mechanism underlying addiction. The growing prevalence of Addictive Disorders highlights the need to identify risk factors and early biomarkers, essential for effective clinical management and improved patient outcomes.

The study by Mrđa and colleagues [6] underscores the growing recognition, in recent years, of the link between MDD and inflammatory pathways. Their study entitled “Association of TNF-α and IL-6 Concentrations with Depression in Patients with Rheumatoid Arthritis” examines the relationship between pro-inflammatory cytokines (TNF-α and IL-6), rheumatoid arthritis (RA) activity, and depression. It included 116 RA patients with depression and 45 patients with primary MDD as controls. Results show that IL-6 levels strongly correlate with RA disease activity and with both Hamilton and Beck depression scores. TNF-α also correlates with depression, though more weakly, and is higher in RA patients. Pain and disease severity were additionally linked to depressive symptoms. The Hamilton scale proved more sensitive than the Beck scale in detecting depression. The findings suggest that TNF-α and IL-6 are independent predictors of depression severity in RA, supporting the role of immune-mediated inflammation in comorbid depression.

Within the context of MDD, Han et al. explored the antidepressant mechanisms of Acorus Tatarinowii in MDD using network pharmacology and molecular docking [7]. In this paper entitled “Exploration of the Mechanisms of Acorus Tatarinowii in the Treatment of Major Depressive Disorder Based on Network Pharmacology and Molecular Docking Techniques”, researchers identified 57 bioactive compounds (notably apigenin, heterotropan, isoelemicin, α- and β-asarone) and 150 intersecting targets between the plant and MDD. Core targets included TP53, STAT3, AKT1, PIK3CA, and PIK3R1. Enrichment analyses highlighted pathways such as PI3K-Akt, cAMP, and MAPK signaling, related to neuroplasticity, inflammation, and oxidative stress. Molecular docking confirmed strong binding affinities of key compounds to these targets. The findings suggest that Acorus tatarinowii may exert antidepressant effects through a multi-component, multi-target, multi-pathway model, supporting its potential as a therapeutic candidate for MDD.

The open-label clinical trial “A comparison of the Treatment Effects of a Risperidone Solution, an Equal Ratio of DHA/ARA, and a Larger Ratio of Omega-6 PUFA Added to Omega-3 PUFA: An Open-Label Clinical Trial” from Yui and Imataka [8] compared the effects of oral risperidone solution (RIS-OS) with two polyunsaturated fatty acid (PUFA) supplementations in 39 adolescents with mild autism spectrum disorder (ASD). Outcomes were measured with the Autism Diagnostic Interview-Revised, Social Responsiveness Scale (SRS), and Aberrant Behavior Checklist. Results showed that RIS-OS significantly improved social motivation and mannerisms compared to both PUFA groups, with no adverse events reported. Plasma biomarkers suggested that reduced ceruloplasmin and increased IGF levels may mediate anti-inflammatory effects linked to clinical improvements. The findings highlight risperidone solution as more effective than PUFA supplementation in improving certain social deficits in ASD.

The last article included in this Special Issue addresses ASD. The review “Neurodevelopmental Impact of Maternal Immune Activation and Autoimmune Disorders, Environmental Toxicants and Folate Metabolism on Autism Spectrum Disorder” from Ayoub [9] examines how maternal immune activation, maternal autoimmune diseases, environmental toxicants, and cerebral folate deficiency contribute to the development of ASD. These factors disrupt fetal neurodevelopment through shared mechanisms, including chronic neuroinflammation, oxidative stress, mitochondrial dysfunction, abnormal synaptic development, impaired myelination, and neurotransmitter imbalance. Evidence shows that infections, autoantibody transfer, pollutant exposure, and folate receptor alpha autoantibodies substantially increase ASD risk. The review highlights the importance of early identification, risk stratification, and targeted prenatal interventions to reduce ASD incidence and improve outcomes.

In conclusion, this Special Issue highlights the broad potential applications of molecular techniques in the study and treatment of a wide spectrum of psychiatric conditions, ranging from neurodevelopmental to affective disorders. We believe that the future of psychiatric care will be inseparable from a translational approach in which peripheral biomarkers, tailored therapies and molecular subtyping of disorders become integral to routine clinical practice, thereby enabling more effective treatments for mental illness. We sincerely hope that these insights may provide readers with valuable perspectives on how such applications could be further extended.
